# Horses are worthy of care: Horse sector participants’ attitudes towards animal sentience, welfare, and well-being

**DOI:** 10.1017/awf.2024.69

**Published:** 2025-01-14

**Authors:** Julie M Fiedler, Margaret L Ayre, Sarah Rosanowski, Josh D Slater

**Affiliations:** 1 Melbourne Veterinary School, Faculty of Science, University of Melbourne, 250 Princes Highway, Werribee, VIC 3030, Australia; 2 School of Agriculture, Food and Ecosystem Sciences, University of Melbourne, Royal Parade, Parkville, Melbourne, VIC 3010, Australia

**Keywords:** animal welfare, animal well-being, equestrian, horse, horse racing, sentience

## Abstract

Organisations for which sentient animals are central to the business model need to demonstrate the safeguarding of animal welfare and well-being. This requires providing positive experiences for animals which is critical to maintaining the social licence to operate. A cross-sectional survey captured the attitudes of experienced horse sector participants regarding sentience, welfare and well-being. Almost all respondents (99.9%; n = 676/677), believed horses were sentient. Analysis of open-ended responses identified two themes: (1) Sentience is a pathway to understanding the mental state, welfare and well-being of horses; and (2) A moral obligation for humans to consider sentience. Respondents’ observations that horses reacted to stimuli and responded to their surroundings underpinned their belief that horses were sentient. Theme one related to respondents’ understanding of sentience and how sentience informed their interpretations of horse behaviours and the making of inferences regarding the equine mental state. Theme two related to a moral obligation believed to exist towards horses because humans manage the horses’ environment and need to consider the impact of their interactions with horses. These obligations were perceived as responsibilities to consider sentience when determining good welfare and well-being in horse activity settings, when interacting with horses and when training and competing with horses. The results suggested a sophisticated understanding of sentience existed among experienced horse sector participants, who recognised the sentient horse as worthy of care. We propose that leveraging experienced participants’ existing knowledge of sentience could support the implementation of the Five Domains model when updating organisational policies.

## Introduction

Sentience, derived from the Latin verb ‘*sentire*’, refers to a conscious animal’s capacity to perceive through the senses and to experience emotions and feelings subjectively (Learmonth 2020). Paul and Mendl (2018) provide an example descriptive definition for emotion based on those used for humans, summarised as the subjective, physiological, neural, and cognitive response to a stimulus or event. It is a conscious experience where the experience is described in terms of arousal (high or low) and valence, as positive or negative, pleasant or unpleasant (Paul & Mendl 2018; Mendl & Paul 2020; Mendl *et al.* 2022). These emotional experiences, or feelings, are labelled with terms such as ‘happy’ or ‘sad’ (Mendl & Paul 2020). Paul and Mendl (2018) go on to argue that when studying comparative sciences and animal emotions, investigators should adopt a prescriptive definition. The authors of this study acknowledge that the terms emotion and feeling are used inconsistently and interchangeably (Kremer *et al.* 2020; Neethirajan *et al.* 2021), and for this reason, both terms, emotions and feelings, appear in this article.

Horses are recognised in law in some jurisdictions, as sentient animals with emotions and feelings, with the capacity to communicate and interact with their environment, other animals and humans (Anonymous 1999, 2022; Mellor 2019; Mellor *et al.* 2020). The sentient horse’s subjective mental experiences, positive and negative, are central to the horses’ perceptions of their welfare and well-being state (Mellor 2019; Littlewood *et al.* 2023). The public is increasingly concerned about the involvement of animals in sports, entertainment, leisure, and tourism (Hampton *et al.* 2020; Hitchens *et al.* 2021; McManus 2022; Vincent *et al.* 2023). This includes activities such as horse-racing and equestrian sports where the sentient horse is central to the business model (Heleski *et al.* 2020; Vincent *et al.* 2023). These activities are open to public scrutiny where, on occasion, perceived threats to animal welfare and well-being, such as the use of the whip on racehorses, may result in concerns being raised (Graham & McManus 2016). Demonstrating intent to safeguard animal welfare and well-being is therefore critical to maintaining organisations’ social licence to operate (Douglas *et al.* 2022; Fiedler *et al.* 2024). In addition to the avoidance of suffering and cruelty, contemporary approaches to animal welfare and well-being incorporate the recognition of sentience and the provision of conditions for positive mental experiences to occur (Mellor 2016; Mellor *et al.* 2020; Littlewood *et al.* 2023). This necessitates that organisations responsible for the governance of animal-related activities continuously review policies and practices to ensure they align with current science and societal expectations for the management of sentient animals (Mellor 2019; Mellor & Burns 2020; Yeates 2022; Williams *et al.* 2023).

Animal welfare and well-being is a complex problem because of the diverse worldviews held about sentience, differing expectations about standards of care, and lack of a universally accepted definition of animal welfare or well-being, all of which act as barriers to change (Linzey 2012; Mellor 2017; Lawrence *et al.* 2022; Mata *et al.* 2022; Haddy *et al.* 2023). Reconciling trade-offs between the organisational, professional, and personal goals of humans and the animals’ requirements may present a challenge for animal-related organisations seeking to remain financially sustainable and retain their social licence to operate (Jones & McGreevy 2010; Fernandes *et al.* 2021; McManus 2022; Edelblutte *et al.* 2023). For clarity, this study adopts the terms welfare and well-being, with welfare as the internal state experienced by a sentient animal (Mellor 2016) and well-being as Simons and Baldwin (2021)’s definition for humans, incorporating a “*state of positive feelings and meeting full potential in the world*”.

This holistic view of welfare and well-being complements the Five Domains model of animal welfare, which provides a validated, systematic framework for assessing animal welfare and well-being, underpinned by the recognition of animal sentience (Mellor 2019; Mellor *et al.* 2020). Organisations, including zoos and horse-racing authorities, are recognising sentience and adopting the Five Domains model to guide updates to animal-related policies and practices (Mellor *et al.* 2015; Anonymous 2020, 2024f; Mellor & Burns 2020). This has wide-reaching consequences for organisations because, with every practice undertaken that involves human interaction with an animal, the potential exists for the animal to experience the situation negatively (McGreevy *et al.* 2018; Mellor *et al.* 2020).

The mental experiences of kept animals are impacted by a variety of conditions, including the proximity, frequency and duration of interactions with humans, human attitudes, and the specific situations in which these occur (McGreevy *et al.* 2018; Mellor *et al.* 2020; Vieira *et al.* 2023). In contrast to many farmed animals, humans are frequently in close proximity to horses. Common examples include training and performance for equestrian sports and regular transport to different activity locations (Spence *et al.* 2017; McGreevy *et al.* 2018; Hogg & Hodgins 2021; Williams *et al.* 2021; Shrestha *et al.* 2022). These activities may involve human-to-horse interactions that might last for extended periods. For example, one study reported that horses in an equestrian centre environment may be ridden for up to four hours daily (Ijichi *et al.* 2023).

Minimum standards for animal management and care are published within laws and voluntary codes, although these tend to lag behind societal expectations (Morton *et al.* 2022; Morton & Whittaker 2022). The approach of animal-related organisations to animal welfare and well-being policy varies but generally includes setting the conditions for animal care, requiring assessment of some or all of the components of welfare and well-being and specifying certain practices (Mellor *et al.* 2015; Mellor & Burns 2020). Activities such as equestrian sports utilise rules to prescribe some practices, for example, stating horses must be presented to officials for veterinary examination (Anonymous 2024b). Other practices may be incorporated within policies, codes or guidance resources, such as the International Federation of Horseracing Authorities (IFHA) transportation welfare guidelines (Anonymous 2023b) or described within educational resources concerning horse management (Anonymous 2013, 2020, 2023a, 2024a).

Demonstrating organisational intent to safeguard the sentient horse’s welfare and well-being requires the recognition of sentience in policy and practice (Mellor 2019; Yeates 2022). However, there is limited research regarding the attitudes of horse sector participants to animal sentience and mental state in the context of organised activities and whether these attitudes may present a barrier or opportunity for effective implementation of sentience-informed policies, practices, and the Five Domains model. This study utilised an online cross-sectional survey targeting experienced horse sector participants involved with horse-racing, sports, riding, and tourism activities to gather their opinions about sentience and its relevance for horse welfare and well-being. The results suggested that a sophisticated understanding of the sentient horse existed in which horses were perceived as worthy of care.

## Materials and methods

### Study description and sampling frame

**Figure 1.**
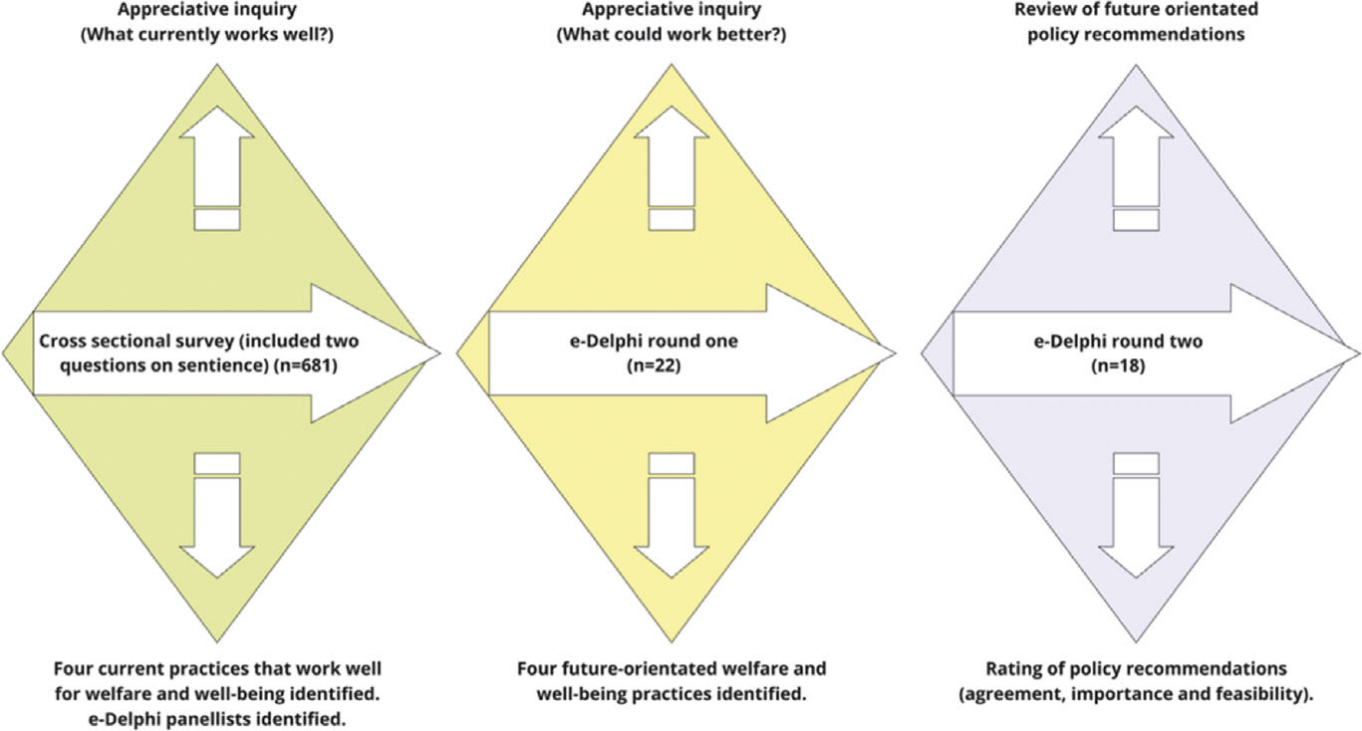
The Futurehorse project: Activities and outcomes. The Futurehorse project is an interdisciplinary mixed-method study investigating horse sector participants’ perceptions regarding practices for horse welfare and well-being. The project utilised two data collection methods: a cross-sectional survey and a two-round e-Delphi. Each diamond represents a data collection point. The survey contained two questions on sentience, the results of which are reported in this study.

This study reports the analysis of a data subset from a larger interdisciplinary mixed-method research project titled ‘Futurehorse’ (Figure 1) that investigated horse sector participants’ perceptions of future-orientated practices for horse welfare and well-being. The Futurehorse project comprised three data collection phases: an online cross-sectional survey and two e-Delphi rounds (Figure 1). The cross-sectional survey contained two questions that called for responses regarding participants’ perceptions of animal sentience, welfare and well-being. The responses to these two questions formed the data subset reported in this study.

The sampling frame was individuals who self-identified as citizens of Australia or the UK, were over 18 years old, had an interest in horse, donkey, or mule welfare, had a minimum of three years’ involvement in horse-racing, riding, sports, or tourism-related activities and were involved in decision-making about horse welfare as an individual or member of a team or committee. The study received approval from the University of Melbourne’s Human Ethics Committee (approval number 2021-21185-19141-6). The survey was uploaded to the University of Melbourne (UoM) platform hosting Qualtrics (Anonymous 2024e). Survey testing was undertaken before utilising the snowball technique for the distribution (Wang & Cheng 2020). The survey URL was promoted via media stories, social media posts, and direct email contact with relevant horse-related organisations. Pre-survey information was provided to respondents, which contained details regarding the study, a consent form, and a link to a published article about the Five Domains model (Mellor *et al.* 2020). The survey was open for 57 days between 5 July 2021 and 31 August 2021. After closing, records were extracted to Microsoft Excel® (Anonymous 2024c) and allocated a unique case number before analysis.

### The conceptual framework

This study was descriptive, recognising that respondents’ perceptions of sentience, the sentient horse, welfare and well-being differ (Charmaz 2014; Rousseau & Billingham 2018). The theoretical perspective of Interpretivism informed the method selection, including the use of the Five Domains model, which is underpinned by the integrated theoretical perspectives of biological function, affective state and natural living. (Charmaz 2014; Mellor 2016; Mellor *et al.* 2020; Stowell 2022; Walker 2023). Interpretivism supported the synthesis and interpretation of respondents’ diverse, subjective lived experiences and perceptions about sentience and horse welfare and well-being, recognising that these are socially, culturally, and historically context-dependent (Charmaz 2014; McGregor 2015). The Five Domains model was utilised as a conceptual framework to guide survey design and for analysis and interpretation (Payne & Williams 2005; Mellor *et al.* 2020). The theoretical perspective informed the discussion of the results and the identification of opportunities and limitations for updating horse welfare and well-being policy and practice.

### Data collection

The data reported in this study were a subset of data collected in an online cross-sectional survey of experienced horse sector participants conducted as part of the larger Futurehorse project. Two questions about sentience were included in this survey; the responses to these questions are the subject of this paper. The first, a closed-ended question, asked respondents whether they believed horses had the capacity for sentience. If respondents answered yes, the survey continued to an open-ended question asking respondents for their opinion on how sentience related to welfare. A definition of the term sentience was provided to increase the clarity of the question and the consistency and reliability of responses (Fowler 2013).

### Analysis

Quantitative data were prepared for analysis by categorising into country, age, gender, equid species of interest, amateur or professional status, job role, and years of experience within the industry. Descriptive statistics were provided as count and percentage. The denominator for each question varied as a result of non-responses. Qualitative data were subjected to pre-coding tasks, which included JMF undertaking initial familiarisation scans, revisiting the research questions, the pre-study literature review, and personal notes (Charmaz 2014; Braun & Clarke 2022). Data were imported into NVivo (Anonymous 2024d,g) to assist with data management. Iterative coding and thematic analysis were conducted in NVivo to identify patterns and unify ideas and meanings (Charmaz 2014; Braun & Clarke 2022). The iterative process involved data clustering and reduction techniques guided by the weighting of the numerical indicators within NVivo (Charmaz 2014; Bogna *et al.* 2020). Group discussions among co-authors supported coding and thematic analysis processes, referring to resources developed by JMF, such as researcher notes and whiteboard logic charts. The co-authors undertook reflexive practices throughout the research process (Beck *et al.* 2021). When reporting the results, de-identified quotes from the raw data were included. Each quote is accompanied by a case number, noted in brackets, which supports the identification of the quote in the raw data set.

## Results

### Demographic characteristics

A total of 677/681 (99.4%) respondents provided an answer to the closed-ended question. Of these, 99.9% (n = 676/677), agreed horses were sentient, and therefore, the survey directed them to the open-ended question asking for opinions regarding sentience and its relation to horse welfare. Of these respondents, most were Australian (n = 614/676; 90.8%), female (n = 483/676; 71.4%) and over 50 years of age (n = 335/621; 53.9%). Almost all respondents were engaged in activities involving horses (n = 658/672; 97.9%), with the remainder engaged in mixed activities involving horses, donkeys and mules (n = 8; 1.2%), exclusively donkeys (n = 5; 0.7%) or exclusively mules (n = 1; 0.1%). In view of this, the term ‘horses’ is used in this study as a collective term that includes mules and donkeys. Most respondents had more than 20 years of experience (n = 590/676; 87.3%), with nearly half identifying as professionals in the horse sector (n = 287/624; 46.0%). There were 592 and 488 complete responses for horse activity and job roles, respectively. Most respondents participated in a horse activity category with a competitive element, such as equestrian sports (n = 286; 48.3%), horse-racing (n = 76; 12.8%) and other horse sports (n = 65; 11.0%). The most common job roles reported by participants included activity manager (n = 218; 44.7%), horse management and/or training (n = 81; 16.6%), and other job roles (n = 81; 16.6%).

### Thematic analysis

The results suggested that an intrinsic understanding of the sentient horse exists among experienced horse sector participants. Respondents believed horses have emotions and feelings and interact with their surroundings, people, and other animals. They made inferences about horse emotions and felt they had ethical responsibilities to care for horses. Analysis identified two main themes. The first theme, ‘Sentience is a pathway to understanding the horse’s mental state, welfare and well-being’ related to respondents’ knowledge about sentience as a means of engaging with the horse’s mental and emotional state and making inferences regarding the horse’s positive and negative experiences. The second theme, ‘A moral obligation for humans to consider sentience’, concerned respondents’ beliefs about the ethical dimensions of interacting with the sentient horse and their responsibility to safeguard horse welfare and well-being.

### Theme one: Sentience is a pathway to understanding mental state, welfare and well-being

The first theme, ‘Sentience is a pathway to understanding mental state, welfare and well-being’, related to how respondents believed horses were sentient with emotions and feelings. This belief was driven by respondents’ observations of horses reacting to stimuli and responding to their surroundings. Respondents made observations of horses’ behaviours that they interpreted and labelled. These suggested that respondents were aware of both a repertoire of instinctive reactions of horses and more complex emotional states, such as a sense of horses feeling safe. These behavioural observations informed respondents’ inferences about mental state and assessments of the horse’s welfare and well-being. Anthropomorphic attributions appeared to assist respondents to connect with the mind and emotions of the sentient horse. Some respondents also raised anthropomorphism as a potential risk to horse welfare and well-being if attributions were improperly applied, which could, for example, lead to horse behaviours being misinterpreted.

Horses were believed to be sentient because respondents observed that horses exhibit behavioural reactions and responses to their surroundings and interactions. They conceptualised the sentient horse as a complex animal whose mental state incorporated both emotional and social needs.

Respondents recognised the sentient horse was *“aware of their surroundings”* [C 947] with an *“ability to respond”* [C 870] to external stimuli and experience *“feelings and sensations”* [C 799]. Behaviours were perceived by respondents as evidence that horses could *“feel emotions”* [C 677] and respond to situations as *“individuals”* [C 185] *“in their own way”* [C 166]. They comprehended horses as a *“flight, fight or freeze animal”* [C 711] who lived in herds, reacted instinctively and were *“attuned to inherent threats”* [C 947] to ensure survival. There was an understanding among respondents that horses’ emotions and feelings can *“effect* [sic] *them and their behaviour”* [C 881] and that they have *“emotional needs”* [C 673] that need to be acknowledged. These respondents explained:

“*They* [horses] *are very good at displaying emotions and reactions to situations*” [C 159: Other horse sports, Amateur].

“*Being sentient means horses have an emotional response to what is happening in their lives”* [C 353: Equestrian sports, Professional].

Respondents also recognised horses as social in their conceptualisations of horse welfare and well-being. They understood that horses were *“social animals”* [C 1139] and perceived a need for them to relate to other horses socially and to benefit from these experiences. For example:

“*Understanding their basic needs as an animal and their emotional and social needs”* [C 218: Other horse activities, Professional].

*“It* [sentience] *has everything to do with welfare, how a horse feels.* [It] *governs its* [the horse’s] *wellbeing physically, emotionally, mentally*, [and] *socially in its herd”* [C 249: Trail riding, packing, driving, Professional].

“*Their psychological wellbeing impacts their decision making and behaviour within the social group”* (C 500: Professional].

Determining that horses were sentient, respondents interpreted behaviours and attributed emotions and feelings to horses. They labelled these using language familiar to human contexts, which may be interpreted as anthropomorphic. Respondents also considered the horse’s mental state, and to what extent these mental experiences were favourable for welfare and well-being.

Some respondents considered that horses may be able to experience *“the full range of emotions* [like] *humans”* [C 28]. Others recognised that a horse *“thinks differently to a human but it does think”* [C 377]. Respondents made inferences about how horses were feeling and perceived these experiences as either positive or negative for the horse. For example, *“pleasure”* [C 935] and *“happiness”* [C 961] were perceived as positive experiences and *“fear”* [C 199] or *“anxiety”* [C 1140] as negative experiences. The perception of *“pain”* [C 108] was mentioned in both physiological and psychological contexts. Respondents perceived horses *“do feel pain physically or emotionally”* [C 746], which were perceived as interconnected. This respondent explained:

“*It’s all about educating humans to realise that horses feel actual pain as well as suffering emotional pain”* [C 756: Welfare, rescue, retraining, Professional].

Respondents considered how these experiences may influence the horse’s sense of welfare and well-being. The concept of welfare and well-being was associated with more positive than negative emotions, feelings and experiences. Experiences perceived as detrimental to welfare and well-being were described using terms such as *“uncomfortable or distressed”* [C 366] or *“upset”* [C 421]. Respondents believed people should provide opportunities for horses to experience situations positively and minimise negative experiences. For example:

“*As horses can feel positive and negative emotions, welfare should be promoted to increase the positive ones”* [C 823: Recreation, Professional].

“*If horses are sentient, they must be able to feel positive and negative affective states and good welfare is when horses feel more of the positive*” [C 936: Other horse sports, Professional].

Respondents discussed horse emotions and feelings in terms of those associated with an innate sense of survival and more complex concepts such as a sense of psychological safety. Safety was, therefore, seen as an interconnected concept, both as a physical environment where horses were safe from injury and as an inferred sense of psychological safety concerning experiences, including those related to interactions.

Respondents recognised the importance of providing horses with a *“safe environment”* [C 629] and *“feel secure”* [C 966] within it. They held a belief regarding the necessity of avoiding situations where horses may *“feel uncomfortable”* [C 1127] These respondents explained:

“*The importance of treating horses with care, compassion and providing a safe environment”* [C 629: Horse racing, Professional].

“*Their environment and feeling of safety is paramount, having major influence over general physical and mental health”* [C 628: Recreation, Professional].

Respondents’ understanding of the sentient horse informed their conceptualisation of welfare and well-being. They perceived an interconnectedness between physical, psychological, and social factors that could influence the horse’s mental experience. Respondents also recognised that by actively monitoring behaviours by considering “*what they* [horses] *are doing*, [and] *how they are living”* [C 497] and interpreting these in terms of feelings, emotions and experiences, they could make decisions and take action to provide opportunities for horses to have positive experiences. These respondents explained:

“*It* [sentience] *helps identify what horses need in regard to welfare*, [by] *monitoring the horse’s response and reaction to changing environments”* [C 371: Recreation].

“*As living, feeling beings they* [horses] *can feel, express and react* [so] *it is important that we learn to understand, to enable horses’ welfare needs are met”* [C 462: Equestrian sports, Professional].

“*If horses are recognised as sentient beings it should allow for greater understanding of their welfare needs”* [C 692: Equestrian sports].

### Theme two: A moral obligation for humans to consider sentience

The second theme, ‘A moral obligation for humans to consider sentience,’ related to respondents’ beliefs that there was a responsibility to first consider sentience in decision-making regarding human-horse interactions, management or training regimes. These obligations encompassed three responsibilities: (1) to consider sentience when interacting with horses; (2) to consider sentience when training and competing with horses; and (3) to consider sentience when conceptualising what is good horse well-being.

A moral obligation to consider sentience was believed to exist because humans have a high degree of influence over the horse’s living environment and the horse’s experiences of interactions with humans. Respondents compared the duty they felt towards horses to their obligations towards themselves and other humans. This belief engendered a sense of ethical responsibility when decision-making about horse management and care and in attitudes towards interactions with them.

Respondents believed there was a moral “*obligation*” [C 339] to exercise a *“duty of care”* [C 502] towards horses, perceiving this *“implies a responsibility”* [C 147] to ensure horses *“enjoy physical and mental comfort and health”* [C 837] because their *“welfare is in our hands”* [C 1152]. They recognised that horses should be *“treated fairly”* [C 1079] and with *“respect”* [C 89], with obligations towards horses considered alongside those for *“ourselves and our friends and family”* [C 445]. These respondents explained:

“*If a horse is aware of feelings and sensations then humans need to provide moral, ethical treatment which equates to prioritising* [the] *horse’s welfare”* [C 978: Equestrian sports, Amateur].

“*They feel emotions and need to be cared for in a way that we care for our mental health”* [C 883: Equestrian sports, Amateur].

“*Horses are sentient beings and have emotions like humans as human wellbeing is important so is wellbeing of the horse”* [C 1134: Equestrian sports, Amateur].

Respondents believed there was a responsibility to consider sentience when humans interacted with horses. They perceived there was a moral obligation to behave empathetically and to be sensitive to the horse’s behaviours. This obligation included monitoring and interpreting behavioural responses to continually inform and adjust how humans interacted and engaged with horses.

Respondents acknowledged that perceptions of sentience could help people to *“understand and sympathise”* [C 962] with horses, making *“humans more thoughtful”* [C 516] and guiding *“how we should relate to them”* [C 982]. They conceptualised interactions as a *“natural relationship”* [C 995] between humans and horses, requiring humans to be sensitive and empathetic. They also felt that humans should be constantly *“reading the horse’s response”* [C 782] to situations and considering horse emotions *“from their perspective”* [C 103]. Respondents believed there was a duty to make informed choices about care and management to ensure optimal welfare and well-being for each sentient horse. For example:

*“How our horses feel about our use is caring about the individual lived experience of our horses*, [their] *mental and physical wellbeing”* [C 185: Other horse activities, Professional].

“*Sentience in relation to horse welfare is about recognising when a horse is showing any adverse or pleasure feelings. This should assist in maintaining optimal wellness for the horse and be identified as a monitoring tool to enhance human-horse interactions. If the sentience is a negative display of feelings, it enables human*[s] *to recognise this response investigate and potentially action a more favourable response to promote horse welfare”* [C 796: Equestrian sports, Professional].

Respondents also believed that horses were emotionally sensitive to *“human wellness and mindsets”* [C 280] and that human *“actions, attitudes, thoughts, and emotions”* [C 897] influenced horse welfare and well-being. Interactions were perceived as relational, whereby horses and humans responded emotionally and behaviourally to each other. These interactive situations were perceived to influence mental experience for the horse, which could be positive or negative. These respondents explained:

“*Horses are extremely sensitive to our actions, attitudes, thoughts, and emotions”* [C 897: Amateur].

“*The ability of horses to feel and recognise emotions in people has an impact on many aspects of their welfare through training practices and everyday handling/animal husbandry*” [C 952: Equestrian sports, Amateur].

Respondents recognised a sense of responsibility to take a sentience-first approach throughout the horse’s life, including in training and competition. They acknowledged that the trainer’s attitude and selection of methods could pose a risk to a horse’s mental state or contribute positively. Incorporating sentience into policy was perceived as a further opportunity to guide expectations about the care and management of horses and safeguard horse welfare and well-being.

There was an awareness among respondents that horse welfare and well-being could be *“compromised in many areas of horse use”* [C 157]. They acknowledged that sentience *“relates to every decision”* [C 946] and all aspects of the horse’s *“living and working conditions”* [C 711]. As sentience was *“fundamentally important”* [C 442] in understanding the horse, they felt it should be *“recognised, considered, understood and worked with”* [C 220] at all times in *“any role humans expect them to perform”* [C 403]. Respondents considered it would be difficult to *“truly manage the needs of the equine”* [C 558] unless there was an understanding of sentience. For example:

“*The environment within which the horse is kept and the handling care around this horse must be targeted with this knowledge of sentience in mind”* [C 836: Veterinary services, Professional].

*“As the animal’s feelings can be heavily affected by its direct and general environment the practices and settings applied by the human element is evidently an important aspect* [of sentience]*”* [C 566: Horse racing, Professional].

Concerns were raised about risks to the welfare and well-being of the horse in competitive situations due to the potentially negative influence of human ambition being prioritised over the requirements of the horse. Respondents commented that sentience was *“not always considered in competitions”* [C 1043] and that prioritisation could result in stress or pain for the horse. This could occur if a horse’s behaviours indicating a negative experience were *“ignored for the sake of a ribbon or prize”* [C 1000]. For example:

“*Horses in competition and training situations are subjected* [to] *stress both emotional and physical from riders and trainers and owners due to unrealistic performance expectations”* [C 1115: Equestrian sports, Amateur].

There was also a recognition that training practices could have *“psychological effects”* [C 349] on horses, perceived as positive or negative. Respondents believed it was important to take the horse’s “*feeling*[s] *into account”* [C 1121], and to choose *“ethical training techniques”* [C 465] to safeguard welfare and well-being. They perceived this involved adopting caring attitudes towards horses, to *“treat them with understanding”* [C 73] and take responsibility for their well-being *“throughout their entire time in our care”* [C 510]. This respondent explained:

“*If we believe that a horse can experience emotions then this should inform our training methods and the activities we ask them to be involved in”* [C 944: Equestrian sports, Amateur].

Attitudes that exhibited *“dominance and control”* [C 72] over horses and training, and practices *“which cause suffering”* [C 343] *“fear”* [C 1140] and *“anxiety”* [C 1136] were perceived by respondents to have no place in a horse’s life, *“any more than* [a] *humans”* [C 273]. There was an awareness among respondents that horses with a poor mental state *“don’t learn”* [C 84] and that negative experiences in one situation *“affect similar future experiences”* [C 188]. They believed that horses should be provided with *“a supportive environment with consistent boundaries”* [C 657] because, with such consideration, horses were perceived to be more *“willing to learn”* [C 976]. There was also a recognition of the need to understand the horse’s *“states of mind”* [C 564] and to select training techniques that involved *“sustainable training, i.e. equitation science”* [C 321]. Respondents associated some horse behaviours with lower levels of stress, using terms such as *“harmony”* [C 769], *“confident”* [C 1083] or *“happy”* [C 380] to describe these. Respondents also perceived that when horses were relaxed, people may be safer around them. For example:

“*It* [sentience] *has great impact. If the horse feels safe and comfortable, he will be able to want to relax and respond*” [C 645: Recreation, Professional].

“*Because of their sentience, the horse’s welfare needs to be optimal for wellbeing, emotional state*, [the] *ability to learn and our safety around them, and enjoyment of, with them*” [C 298: Equestrian sports, Amateur].

Respondents acknowledged that in some circumstances, horse carers may not be aware that their actions could be considered abuse. These practices, such as suffering through *“ignorance”* [C 1042] or a *“lack of respect or neglect”* [C 779], may be recognisable or *“not necessarily be openly evident”* [C 872] to persons responsible for horses. In other situations, abusive practices could be normalised, and individuals unaware of the consequences. For example, respondents considered that repeated negative experiences, likened to *“bullying horses”* [C 688], could lead to horses no longer trying to respond, a behaviour labelled as *“learned helplessness”* [C 14]. They believed that better well-being could be ensured if expectations for its provision were written down in policy. Embedding sentience into policy was considered necessary for *“enforcing and creating animal welfare legislation”* [C 960] and other types of guidance, such as standards of care. These respondents explained:

“*Animal sentience is one of the most important welfare definitions and is most often overlooked in horse sports*” [C 416: Equestrian sports, Professional].

*“The standards of care within industries should reflect* [an] *awareness that horses have feelings”* [C 354: Other horse sports, Professional].

Respondents believed there was a responsibility for themselves, other individuals, and organisations to consider sentience when conceptualising what is ‘good’ horse welfare and well-being and to ensure its provision. Welfare and well-being were perceived as a holistic, positively oriented concept extending beyond avoiding harm or providing minimum standards of care. Respondents also perceived welfare and well-being also perceived welfare and well-being as a life for the horse which held value and could be judged by humans as being of a good quality, based on inferences made about the horse’s mental experiences. For example:

“*Sentience is the foundation on which good equine welfare is built”* [C 308: Professional].

*“It* [sentience] *relates to their* [the horse’s] *holistic quality of life and wellbeing”* [C 161: Amateur].

Respondents awarded value attributions to the sentient horse’s lived experiences. These were perceived in general terms, such as *“good horse welfare”* [C 482] or *“high levels of welfare”* [C 775] and, in more specific terms, relating to the quality of the horse’s lived experiences, such as *“a life worth living”* [C 161]. These respondents explained:

“*Establishing or understanding an animal’s signs of negative and positive emotions can help ensure that their mental wellbeing is safeguarded so that they can experience a life worth living”* [C 583: Recreation, Professional].

“*I define good horse welfare as a life worth living. Sentience is the mechanism to gauge this”* [C 555: Horse racing, Professional].

“*It’s our duty to keep and make use of horses in ways which allow them the highest possible quality of life”* [C 251: Horse sports, Professional].

Respondents believed that sentience informed a sense of moral duty towards horses, including taking responsibility to *“reduce or eliminate their suffering”* [C 115]. They associated abuse, suffering and cruelty with harm, and *“lower than ideal welfare levels”* [C 378]. Respondents perceived that horse “*abuse*” [C 789] was a *“result of a human’s actions”* [C 722] and decisions and that suffering was *“not acceptable”* [C 833]. For example:

“*Suffering through neglect, cruelty or ignorance is unacceptable because they* [horses] *are aware and feeling”* [C 1042: Horse racing, Professional].

“*Welfare is inextricably linked to sentience as it defines the severity of actions against an animal based on how they feel”* [C 999: Equestrian sports, Amateur].

*“Recognising that living beings, other than humans, feel and process those feelings in the context of themselves must lead humans to ask: what is harmful? what should we not do? what should we do better? what freedoms should be accorded sentient beings?”* [C 144: Trail riding, packing, driving, Professional].

Respondents believed they had an obligation to relieve horses of suffering and expressed concerns about horses who lived with pain. They considered there was a duty for horse carers and owners to manage forms of physical, emotional, or social pain. For example, in cases of untreatable physical pain, it may be appropriate to consider *“end-of-life options”* [C 94], perceiving *“euthanasia as a release”* [C 551] from suffering. Pain management extended to forms of emotional and social pain, for example, *“loneliness, grief”* [C 711], *“separation anxiety”* [C 1139], and *“distress”* [C 643], requiring an awareness of a horse’s “*physical and psychological*” [C 745] requirements and “*preferred social environments*” (C 32). These respondents explained:

“*If horses are feeling pain, they should be treated appropriately. Serious injuries are often very obvious, and most people can arrange veterinary treatment. Emotional pain is not well understood, and there are many horses I see that are started on the animal helplessness path, and owners simply do not realise this”* [C 196: Trail riding, packing, driving, Amateur].

“*The actions people take around horses include riding how they are kept, exercised, etc, need to be in such a manner they do not cause mental or physical pain for a horse and if they are in pain should be such* [that], *they work to get rid of that pain”* [C 1178: Trail riding, packing, driving, Amateur].

## Discussion

This study aimed to investigate the attitudes of experienced horse sector participants towards horse sentience, welfare and well-being. The results indicated that survey respondents had an insightful comprehension of sentience, that humans had responsibilities towards the sentient horse and that horses were worthy of care. Analysis identified two themes, the first relating to sentience as a pathway to understanding mental state, welfare and well-being, the second relating to a moral obligation for humans to consider sentience when caring for the horse.

The demographic profile of almost all survey respondents in this study was older, experienced females. This was consistent with previous studies that utilised online surveys to collect data from horse sector participants and with studies surveying this sector regarding attitudes toward horse emotions (Dashper 2017; Hötzel *et al.* 2019; Spence *et al.* 2019; Chapman *et al.* 2020; Fletcher *et al.* 2021; Luke *et al.* 2022).

Theme one related to respondents’ understanding of sentience, the conceptualisation of the sentient horse, and how sentience informed interpretations of behaviours and inferences of emotional state. These fundamental elements shaped perceptions about horses as individuals and each horse’s welfare and well-being state. There was a belief that horses feel pain and interact, communicate, have experiences and perceive their welfare and well-being state. These were all features of a sentient animal as described by Mellor (2019) and reported in studies about animal carers’ recognition of emotional states in cattle (Schuppli *et al.* 2023), birds (Kleinberger *et al.* 2023), dogs and cats (Pickersgill *et al.* 2023), and in horses (DuBois *et al.* 2018; Hötzel *et al.* 2019; Fletcher *et al.* 2021; Tolls & Carr 2021). In this study, respondents recognised that behaviours, personalities and temperament traits were unique. The results align with studies that report characteristics unique to individual horses, such as behaviours, personality and temperament traits and food and social preferences (Hausberger *et al.* 2004; Van Den Berg *et al.* 2016; Ladewig 2019; Jolivald *et al.* 2022; Jaramillo *et al.* 2023; Kieson *et al.* 2023).

The study results showed that respondents possessed a holistic and sophisticated understanding of horse welfare and well-being, including recognition of horse sentience, emotions and mental experiences, and the traditional focus in horse care and management practices centring on physical health measures and living conditions (Green & Mellor 2011). This suggests that there may be a shift in thinking and practices that build on the provisions-focused Five Freedoms paradigm towards recognising the requirements of horses as outlined in the animal-centric Five Domains model. Animal health, welfare, and well-being assessments have historically focused on objective measures relating to physical health and living conditions (Duncan 2006; Mellor 2016). The current study suggests that respondents have moved towards recognising subjective animal mental experiences, which are inherent in the Five Domains model (Mellor *et al.* 2020).

Another aspect identified in Theme one related to respondents’ perceptions of a horse’s sense of physical and psychological safety. These interconnected concepts concern physical safety related to hazards in the horse’s living or activity environment that might cause injury, whereas psychological safety is related to an inferred mental state of the horse. This study found that respondents connected inferences of the horse’s sense of safety with positive mental experiences, which was therefore perceived as beneficial for horse welfare and well-being. The results resonate with animal science, recognising an animal’s interconnected sense of physical and psychological safety (Mellor *et al.* 2020; Leconstant & Spitz 2022; Littlewood *et al.* 2023).

Safety-related studies in the horse sector often consider the physical safety of humans around horses or the horse’s physical health (Hitchens *et al.* 2019; Chapman *et al.* 2020, 2022). To date, how horse sector participants infer a horse’s sense of psychological safety in a range of situations remains understudied. Future research should investigate human perceptions of personal safety and the making of inferences about the horse’s sense of safety. The results could inform updating human workplace safety and animal welfare and well-being assessment frameworks.

Theme two related to respondents’ sense of moral obligation to consider horse sentience and the horse’s mental state during interactions and when making decisions about management, training, or performance regimes. Sentience also informed respondents’ understanding of horse welfare, well-being and quality of life. There was a belief that a moral obligation existed for humans to consider what constituted good horse welfare and well-being, and to provide the conditions for the horse to have experiences inferred as positive. Respondents’ sentience-informed reflections on welfare and well-being incorporated ethical perspectives about doing what they felt was right or good for horses. They awarded the horse’s lived experiences a value, or worthiness, which was perceived in aspirational terms, such as a life that was worth living (Heleski & Anthony 2012; Mellor 2016; Littlewood *et al.* 2023). Respondents conceptualised a value-spectrum of the horse’s lived experience, with the avoidance of suffering counterbalanced with worthiness or quality-related values. The spectrum not only orientated the individual’s understanding of welfare and well-being but facilitated communication of these concepts to others.

Another aspect of Theme two related to respondents’ belief that understanding sentience could influence a human’s choice regarding their attitudes and behaviours when interacting with horses. They perceived human-horse interactions as relational and that horses were responsive to human mindsets and emotions. Therefore, attributes such as compassion, empathy and sympathy were preferable when interacting with horses, as they believed these attributes could contribute positively to horse welfare and well-being. These beliefs resonate with Domain 4 of the Five Domains model, which refers to empathetic attitudes as a positive human attribute (Mellor *et al.* 2020).

Respondents identified the need to show compassion and to act with intent to shape human-horse interactions to provide the conditions for positive experiences for the horse. These findings align with previous research calling for a welfare and well-being culture in horse-related activities that supports ethical human-horse interactions because the sentient horse’s experience also matters (Dashper 2017; Hogg & Hodgins 2021; Campbell 2023). The findings also align with studies regarding the management of working equids (Cousquer 2023; Haddy *et al.* 2023), reinforcing the universal importance of considering sentience in all equid welfare and well-being contexts. More broadly, training approaches for kept animals are moving away from a reliance on attitudes and techniques that focus on dominance and avoidance of suffering towards the recognition of positive mental experiences and a sense of mutualism (Anonymous 2017; Brando & Norman 2023; Cousquer 2023; Fernandez 2023).

Within Theme two, the ethical perspectives of respondents were interpreted to encompass the concept of animal safeguarding. Adapted from the human context (Anonymous 2024h; Fiedler *et al.* 2024), animal safeguarding as a collective, active model of community-wide care is still emergent in the horse and wider animal sector (Fiedler *et al.* 2024). If safeguarding is to be realised, all individuals with decision-making about, or influence on, a horse’s welfare and well-being state should aspire to identify opportunities for the horse to gain from interaction with humans and to ‘flourish’ in the home stable and competitive environment (McLean & McGreevy 2010; Javanaud 2022; Campbell 2023; Fiedler *et al.* 2024). Ensuring that positive experiences for horses outweigh negative experiences and formalising that aim in management and training principles and when competing would be a significant shift from previous approaches to animal welfare policy (Mellor 2016; Yeates 2022; Littlewood *et al.* 2023).

In this study, observations and interpretations of horse behaviours informed the inferences made by respondents about a horse’s emotional state. Respondents did not use the term inference, nor were detailed descriptions provided regarding the conscious process of making these. However, this study adopts the term, acknowledging that the process occurs in both scientific and social contexts (Polanyi 1966; Schuurman 2017; Mellor 2019). In both contexts, the results of research, including equine ethograms and knowledge about equine senses and perceptual abilities, can be utilised to support the making of inferences about feelings, such as those associated with pain (Gleerup *et al.* 2015; Rørvang *et al.* 2020; Ladewig *et al.* 2022; Bradshaw-Wiley & Randle 2023). In this study, various inferred emotions and feelings were reported, such as calmness or fearfulness. These results align with studies reporting that companion animal owners, farmers, zookeepers (Dawson *et al.* 2019; Webber *et al.* 2020; Schuppli *et al.* 2023) and horse sector participants (Hötzel *et al.* 2019; Bornmann *et al.* 2021) infer animal emotions.

### Alignment framework – a practical approach

Gaining insight into experienced horse sector participants’ conceptualisation of the sentient horse could assist organisational approaches to improve horse welfare and well-being. The current study has specific implications for horse-related organisations because activity participants may expect organisations to recognise sentience and commit to safeguarding horse welfare and well-being. Any misalignment between participant expectations and organisational policy and practice could impede the implementation of the Five Domains model and might reduce public trust in the organisation’s capacity to safeguard equine welfare and well-being. Potential barriers to adopting sentience-informed policies and practices within an organisation could be overcome by demonstrating where participants’ viewpoints align with the current scientific evidence. This could be achieved through an Alignment Framework (Table 1), which can be used as a checklist to support processes for developing or reviewing policies related to horse welfare and well-being (Edelblutte *et al.* 2023). The first column, the checklist, proposes three individual but interconnected elements, informed by the study results and referenced by literature. The second column incorporates results from this study, and the third column provides examples of literature from science, philosophy, and ethics. It also provides evidence for the statements (column 1) and viewpoints of horse sector participants (column 2). By demonstrating how participants’ viewpoints and animal welfare science, ethics and philosophy can be aligned through the four statements, the proposed framework could act to engage wider organisational stakeholders in the topic of sentience and as a tool to assist with the implementation of the Five Domains model and to improve associated policies and practices.

**Table 1.** A proposed alignment framework for horse-related organisations to guide the development of sentience informed policies to promote positive horse welfare and well-being. The framework provides an evidence base for stakeholder engagement through a checklist that aligns the results from this study with relevant examples from the literature.

Alignment framework for organisations: Sentience			
No	Checklist	Horse sector participant understanding (Results from this study)	Science, philosophy and ethics (examples)
1	Horses are sentient.	Recognising that horses are sentient, possessing emotions and feelings.	Sentience, pain (Mellor 2019; Mellor *et al.* 2020; Edelblutte *et al.* 2023). Perceptions relating to equid sentience, pain, and emotions (DuBois *et al.* 2018; Hötzel *et al.* 2019; Bornmann *et al.* 2021; Fletcher *et al.* 2021; Tolls & Carr 2021). Policy (Yeates 2022).
2	Horses are individuals.	Recognising sentient horses as unique individuals.	Horses are individuals with unique temperaments, personalities, preferences, and behavioural characteristics (Hausberger *et al.* 2004;Van Den Berg *et al.* 2016; Mellor 2019; Edelblutte *et al.* 2023; Haddy *et al.* 2023; Jaramillo *et al.* 2023; Jolivald *et al.* 2023; Kieson *et al.* 2023).
3	Horses are social and communicate.	Perceiving the sentient horse as social. Providing opportunities for social connection.	Equine sociality (Rørvang *et al.* 2018; Mellor 2019; Briard *et al.* 2021; Maeda *et al.* 2021; Edelblutte *et al.* 2023; Harvey *et al.* 2023). Perceptions about equine social factors (Birke & Thompson 2018; Hötzel *et al.* 2019; Bornmann *et al.* 2021; Merkies & Franzin 2021).

### Study limitations, future research

It is important to acknowledge that this study is qualitative, employing thematic analysis methods, which are inherently subjective (Charmaz 2014; Braun & Clarke 2022). A validated sampling frame for the UK (Spence *et al.* 2019) or Australian horse owners was not known to exist when this study was conducted. Respondents were therefore invited to participate in the survey via a link primarily promoted on social media. Participants who chose to participate may therefore have had pre-existing views on the topic, which may have introduced bias. In the demographic section of the survey, respondents were asked an open-ended question about their industry sector, as opposed to being asked to choose from a categorical list of industry sector types, because we did not wish to constrain responses. The design of this question meant that respondents used a wide range of sometimes inconsistent terms to describe their industry sector which complicated *post hoc* categorisation. As a result of this, responses about which industry sector respondents participated in were clustered into broad categories. It is therefore difficult to determine how representative the sampling frame of this study was of the industry as a whole. Participation was opt-in, with respondents self-assessing as having three or more years of experience in the sector and interested in horse welfare. Therefore, the views of inexperienced individuals or individuals who were not interested in welfare were not captured. The survey described sentience as ‘the capacity for an animal to feel and experience emotions.’ In designing this study, the authors identified that providing a short definition of sentience would enhance respondent understanding of the question (Fowler 2013). The approach may have added response bias by providing context beyond what participants may have already understood as the definition of sentience. However, the authors considered that the breadth and depth of responses indicated that the definition did not unduly influence the quality of the responses. Methods employed to manage risks associated with subjective interpretation included the adoption of reflexive practices and utilising the Five Domains model as the overarching framework for theme development (Payne & Williams 2005). The study’s conceptual framework, incorporating the Interpretivist theoretical perspective, should assist with approaches to transfer the qualitative results to other situations and settings, supporting the generalisability of this work (Payne & Williams 2005; Carminati 2018). Further research is needed to describe sentience-informed practices for organised activities in competitive and non-competitive contexts.

### Animal welfare implications

Acknowledging animal sentience is integral to providing the conditions for animal welfare and well-being (Mellor 2019). Horse sector participants’ attitudes towards sentience inform practices for the management and care of horses. It is important to gain an insight into these attitudes because many horse-related practices originated in an era when horses were used for war and transport (McLean & Christensen 2017), and before sentience was recognised widely in society. As this study proposes, updating policies and practices to recognise sentience will ensure that organisations remain aligned with current science and public expectations for safeguarding horse welfare and well-being.

## Conclusion

This study explored the perspectives of experienced horse sector participants concerning animal sentience, welfare and well-being. The results suggested that sector participants had a sophisticated understanding of sentience and believed the sentient horse was worthy of care. A moral obligation to consider sentience when interacting with horses, including when training and in performance situations, was identified. It is proposed that by leveraging the knowledge of experienced horse sector participants and implementing the Five Domains model, an improvement in the quality of life of horses in organisational contexts is achievable. This could be facilitated by a framework that aligns knowledge from science, philosophy and ethics with the opinions and beliefs of equine sector participants. An example is provided in this paper to demonstrate how the results of this study can be aligned in this way, potentially enabling horse sector participants’ understanding of sentience to act as a resource to engage activity participants in the topic of animal sentience. Future research is needed to define and describe sentience-informed management, training and performance practices for organised activities such as equestrian sports and horse-racing. While there may be a cost to introducing the recognition of sentience into policy and practices in organisations where animals are central to the business model, the cost of not doing so will be greater if the social licence to operate is lost.
